# The role of digital readiness innovative teaching methods in music art e-learning students’ satisfaction with entrepreneur psychological capital as a mediator: Evidence from music entrepreneur training institutes

**DOI:** 10.3389/fpsyg.2022.979628

**Published:** 2022-09-08

**Authors:** Ye Huang

**Affiliations:** College of Arts, Xiamen University, Xiamen, China

**Keywords:** digital readiness, e-learning satisfaction, mindfulness, psychological capital, innovation

## Abstract

The way of our living and working has changed intensely throughout the past half-century. The era we live in is interlinked with rapid technological changes, paving the way for digitalization. The students are considered digital natives and are expected to have e-learning abilities to improve their academic effectiveness. However, digital readiness is an important factor that can play a valuable role in boosting students’ e-learning abilities and satisfaction. The previous studies of students’ e-learning abilities revealed the lack of students’ digital readiness for academic achievements. Therefore, the present study aims to examine the role of digital readiness in the e-learning satisfaction of students. Based on the theory of motivation, the present study attempts to check the association of digital readiness with e-learning satisfaction. The current study also determines the relationship of digital readiness with entrepreneur psychological capital. Further, this study examines the correlation of entrepreneur psychological capital with e-learning satisfaction. The present study also assumes the mediating role entrepreneur of psychological capital and moderating role of mindfulness. For empirical analyses, this study gathered data from 376 music learning students of entrepreneur training institutes in China through a structured questionnaire method using a convenient sampling technique. This study applied partial least square structural equation modeling for empirical analyses using Smart PLS software. The present study confirmed that digital readiness positively correlates with e-learning satisfaction and psychological capital. The findings also acknowledged that psychological capital positively enhances e-learning satisfaction. The results also confirmed that psychological capital mediates the association between digital readiness and e-learning satisfaction. However, the outcomes revealed that mindfulness does not moderate the association between digital readiness and e-learning satisfaction. On the other hand, the findings acknowledged that mindfulness moderates the relationship between psychological capital and e-learning satisfaction. In addition, this study’s findings also serve the literature by providing important theoretical and practical implications. This study points out that digital readiness is an important antecedent to increasing students’ learning satisfaction and performance. The findings also suggest that students’ mindfulness could play a bridging role in enhancing their performance.

## Introduction

Institutes of higher education have been striving hard to ensure quality education with the use of innovative technologies. One aspect of this particular struggle revolves around the idea that innovative technology should be used in a way to enhance the delivery of quality education (Deng and Tavares, 2013). There has been a vast prevalence of e-learning environments at educational institutes. The e-learning ecosystem assists in delivering educational resources to the concerned people, enhance teacher-student communication buildup around learning and help people track their progress, etc., (Islam, 2013). E-learning at higher educational institutes not only ensures sustainable learning improvement but rather tends to inculcate skills and learning experiences that can last a lifetime. After all, the process of acquiring education or acquiring particular skills is not limited to academic success but it is to solve real-world issues by successful application of these skills. Therefore, the e-learning ecosystem at an academic institution is based on the principle to assist and improve the teaching and learning practices with the use of innovative technology-enabled tools and methods (Eze et al., 2018). E-learning has a number of benefits such as it reduces the cost of physical teaching and learning infrastructure, makes it easier to create and share course content, helps in making the educational content readily available at any time or place and integrating global educational network (Pham et al., 2019). Recently, the focus of learning has shifted to an extent from teacher-centered to student-centered learning particularly because of the primary design or scheme of the e-learning ecosystem at university campuses. Student-centered learning makes sure that the students are satisfied with digital learning experience (Ituma, 2011). The digital learning experience requires digital readiness of the students. Digital readiness is defined as behavioral competencies vis-à-vis the process of digital transformation. It is a measure of combination of cognitive skills, digital literacy and digital proficiency of the students (Hong and Kim, 2018). The digital skills, e-learning attitudes and knowledge of digital technologies define the digital readiness of the students. It is a key to success and satisfaction of students in an e-learning experience (Roffe, 2002). Previous research studies have shown that the students who are digitally literate and show digital readiness have shown better academic outcomes (Hong and Kim, 2018).

Student satisfaction with e-learning methods depends on various factors including innovative teaching methods being used by teachers (Cheng et al., 2021), student mindfulness during the whole process of learning (Hafeman et al., 2020), and the psychological capital of the entrepreneur of the institutes (Luthans et al., 2007a). The use of innovative teaching methods requires teachers to present themselves digitally ready and equipped with innovative teaching skills (Cheng et al., 2021). From preparing educational material to delivering it to the learners, from regulating courses to ensuring sustainable learning, e-learning encompasses the use of technology-enabled tools and methods to ensure a wholesome learning experience for the students at institutes of higher education (Parkes et al., 2015). With significant advancements in multimedia and network technologies, e-learning has immensely benefitted. High-speed internet, smart technological devices, high-quality video and learning management systems driven by artificial intelligence are the core factors that have made e-learning the norm of the day (Cidral et al., 2018).

In-person technology-driven instruction was deemed as quite simple in comparison to the recent phenomenon of highly complex and integrated teaching-learning experience/practice. Lectures can be attended and even recorded remotely, online chatting platforms assist in the instant exchange of ideas, discussion boards have diminished distances and online services of social networking have become a household name. All these technology-driven and –enabled tools to ensure blended learning that has aptly adopted by the institutes of higher education as there has been irrefutable evidence that learning has surely become efficient and convenient with the use of modern e-learning tools (López-Pérez et al., 2011).

There has been a mixed result as far as students’ success and e-learning satisfaction are concerned keeping in view the adoption of these e-learning systems. An increased level of satisfaction among students vis-à-vis the learning experience has been observed (Lyons and Evans, 2013). Moreover, there has been a decrease in the number of school dropouts, academic performance has also been improved, whereas, critical and reflective thinking has also been observed as increased among students (Saadé et al., 2012). On the other hand, many studies also observed that the connection between the use of e-learning systems and students’ performance has no link at all. Roffe says that the reason behind such mixed results could be the level of expertise or knowledge that the students have in using e-learning to the best of its use. Furthermore, factors such as individual characteristics, general attitude, level of confidence in using e-learning systems and digital readiness could also be the reason why some students did not show any improvement in their studies despite the availability of e-learning platforms.

The e-learning satisfaction of the students depend upon many factors. This study also aims to study the relationship of psychological capital with the e-learning satisfaction (Castillo-Merino and Serradell-López, 2014). By its definition, psychological capital is linked with positive psychology and it focuses on individual’s strengths rather than weaknesses. It is a combination of four factors including self-efficacy, optimism, resilience, and hope. Psychological capital enhances the entrepreneurial competencies, learning and skills of the students (Alessandri et al., 2018). Besides digital readiness and psychological capital this research study argues about the moderating role of mindfulness between digital readiness and e-learning satisfaction, and psychological capital and e-learning satisfaction. Therefore, it is important to understand the concept of mindfulness. According to American Psychological Association mindfulness refers to the ability to objectively focus on a particular aim. It is the ability to comprehend and accept an untoward occurrence. Mindfulness can increase psychological capabilities of an individual. The study focuses on the element of mindfulness and its impact on the learning process (Hafeman et al., 2020).

While various researchers have already studied digital readiness, e-learning satisfaction, mindfulness and psychological capital individually, the relationships among these variables have not been yet explored in a single study. Thus, the current study is based on the research that contributes to answering the following research questions: First, how digital readiness is associated with e-learning satisfaction and psychological capital of the entrepreneur of a music institute? Second, how psychological capital is associated with e-learning satisfaction? Third, what is the role of psychological capital between digital readiness and e-learning satisfaction? Fourth, what is the role of mindfulness between digital readiness and e-learning satisfaction and psychological capital and e-learning satisfaction? The current study aims to fill the available literature gap by finding answers to these research questions. The theory of motivation provides the fundamental theoretical support for this research study. Based on previous research studies explored through an extensive literature review the study assumes that digital readiness has a positive association with e-learning satisfaction and psychological capital. It also assumes that psychological capital, which is linked positively with e-learning satisfaction, also mediates the positive association between digital readiness and e-learning satisfaction. Furthermore, the study hypothesizes that mindfulness moderates the positive association between digital readiness, psychological capital and e-learning satisfaction. These hypotheses were further studied by using five points Likert scale. The data for the empirical analysis of the study was collected through questionnaires.

The current study has significant theoretical as well as practical implications, which provide useful and practical suggestions to educationists, entrepreneurs, scientists and students. The further assembly of the article follows hypotheses development *via* literature review, scientific data analysis and interpretation. The article is closed with a discussion, conclusion, implications and limitations of the study.

## Literature review and hypotheses development

### Theoretical background

According to motivational theory, those students who actively remain engaged in the usage of e-learning systems tend to exhibit better outcomes in terms of learning. The process of learning in students is a highly complex matter as students are supposed to be thought of as sponges that can absorb the information provided by teachers, articles or books, etc., rather the process of learning encompasses interaction, open discussion, free discourse and knowledge application etc., (Bryer and Seigler, 2012). Therefore, according to the theory of motivational theory psychological capital (PsyCap) is more responsible for enhancing the learning ability of students, therefore, students can be empowered by giving them a social learning environment. Furthermore, the students with a high level of PsyCap are better placed to benefit and learn using online curricula as they have a deep sense of self-efficacy, a higher level of motivation, a sense of optimism and a strong will to achieve their targets that ultimately enhances their chances of learning.

E-learning is defined as delivery of instructions through technological tools with an intention to support the process of learning (Clark and Mayer, 2012). There has been a concern around the quality aspect of online learning right from the start. The established educational community in particular and society, in general, have expressed these concerns that online learning cannot attain the level of quality that offline learning ensures (Akdemir and Koszalka, 2008). Because of these concerns student often gauge their learning experience through the contest of satisfaction (Bolliger, 2004). Since contemporary students think of information as an exchangeable commodity, therefore, this very perception regarding information makes them think that collaboration is also a significantly fundamental learning outcome (Dziuban et al., 2013).

The boundaries of a traditional classroom are being changed rapidly through technology (Shirky, 2009). The avenues of communication have become significantly varied and students tend to collaborate with their teachers and peers using all kinds of different communication mediums for the sake of learning and sharing (Norberg et al., 2011). The instructor’s role has been changed drastically because of the adoption of blended systems of learning (Liu and Hwang, 2010). Students also have different learning preferences such as synchronous or asynchronous which has also impacted the role of the instructor in the contemporary learning environment. New, improved and authentic assessment techniques are required for modern-day students and at the same time, it has also been focused that what actually moderates the satisfaction and expectations of students in their process of learning. Online education opportunities have, according to some studies, reduced the level of indecision of students toward formal education (Long, 2011). Active learning has been preferred by many students instead of passive learning as it is deemed that real-world interaction always demands skills that encompass active problem-solving tendencies. Therefore, students expect to have the same kind of experience in the class as well during the process of learning (Dziuban et al., 2003). As contemporary students require more and more outlets for creativity and collaboration and that is conveniently provided by the e-learning ecosystem that comes equipped with a vast variety of such outlets.

### Digital readiness and student satisfaction

Digital readiness is defined as behavioral competencies vis-à-vis the process of digital transformation. It also included cognitive skills and digital proficiency (Hong and Kim, 2018). Digital readiness is of tremendous importance in today’s learning space. Digital readiness can be achieved through having knowledge of technology, skills to use technology and competency to use e-learning outlets to meet educational aims (Hong and Kim, 2018). Skill, competency and tendency to utilize technology have been seen as basic factors in improving the learning abilities of the students (Jones, 2012). A study claims that Korean students may or may not effectively utilize their knowledge of technology to enhance their learning ability as they did not always associate their tech-savviness with academic activities (Hong and Kim, 2018). There has also been a wide gap between the use of technology by students for their leisurely pursuits and academic endeavors (Margaryan et al., 2011). For being able to digitally ready, students are required to learn meaningful use of technology for academic work. The students who put technology to work by active participation in the digital process of learning and can do a critical evaluation of digital culture around learning are more prone to have better academic outcomes.

Students’ satisfaction is defined through various approaches. Research on Community of Inquiry (Garrison et al., 1999) defines learning experience in terms of social, cognitive and teaching presence that can lead to satisfaction of students (Rubin et al., 2013). The research determined that features of a learning management system have a profound impact on the community’s perception as per the framework provided by the Community of Inquiry. Another study contends that it is the teaching presence that holds the central importance in students’ perception regarding online learning. Furthermore, the role of the interaction construct is also important whether it is face-to-face learning or otherwise (Kuo et al., 2013). As a matter of fact, many studies have shown that the level of satisfaction vis-à-vis any learning environment remains highly correlated to the quantity as well as quantity of interactions during the process of learning. At the same time, it has also been contended that factors such as demographics and culture are also important in designing the appropriate channels of interaction, particularly in an online learning environment.

Five elements of satisfaction were identified by Ke and Kwak (2013). Three of these are related to the learner while the other two are related to the process of learning. The relevance of the learning material to the learner, the level of autonomy of the learner and the learner’s readiness for the use of technology are the three elements that are related to the learner. Authentic learning and active learning are the other two elements identified in the research. The combination of learner’s efficacy toward technology and learner’s interaction with the instructor as well as the content determine the student’s satisfaction as far as online learning is concerned (Kuo et al., 2013). However, the criterion approach was used by Battalio (2007) according to which effective interaction between the learner and the instructor are required for a positive rating of the course. Another study shows that an instructor’s choice of different online tools to conduct an online class is influenced by students’ expectations (Keengwe et al., 2012). The study concludes that satisfaction remained most impacted by the convenience of learning combined with the level of effectiveness of online tools being used.

A study found six elements that contribute to the satisfaction level of learners (Dziuban et al., 2019). Success is correlated to satisfaction in a learning environment and it becomes even more pertinent in an e-learning environment. Satisfaction keeps students engaged, motivated and receptive making the entire learning environment effective. The students who are either dissatisfied or ambivalent have an effect on the entire learning environment making it complex and difficult for the instructor to create an engaging and meaningful learning atmosphere that can help dissatisfied learners. Such circumstances give rise to difficult interactions between the faculty members and the students. This can also incorrectly lead teachers to believe that the dissatisfied students have trouble learning *via* online modalities (Dziuban et al., 2019).

It is proving to be elusive to precisely identify learners’ satisfaction with online learning since that can be highly dependent on a context. However, if the individual constituencies of the students are probed the issue of defining satisfaction seems much less daunting. These definitional issues, according to Watts (2011), arise from confirmation bias. Watt contends that we tend to accept information that is in accordance with our beliefs more readily than the information that goes against our beliefs. Keeping confirmation bias in view, students tend to react to only those things that they expect such as their already established assumptions about the course. However, when the student encounters a discrepancy between their expectations and reality they tend to become ambivalent by having both positive as well as negative feelings. These are called mixed emotions by Long (2011).

The validation of the Community of Inquiry (COI) framework was carried out through factoring by Arbaugh (2007) while incorporating cognitive, social and teaching presences. Furthermore, research by Stewart, Hong and Strudler used principal component analysis to understand students’ satisfaction regarding online learning. The research found quite a complex dimensionality of varying levels of student satisfaction (Stewart et al., 2004). The evaluation of the online learning modules was based on issues such as the outlook of the web pages, usage of hyperlinks, technical issues using online learning modalities, teaching techniques, lecture delivery and general online academic interaction. According to Bangert (2006) there were four basic elements to evaluate online courses. These elements were interaction, active learning, task-time and learner’s cooperation (Bangert, 2006). Later on, in another study, he validated his earlier study by using confirmatory and exploratory factor methods (Bangert, 2008).

Instead of identifying underlying dimensionality, another research used classification and regression trees to asses learners’ assessment of online academic content. The research used three constructs such as facilitation of learning, instructor’s ability to inculcate knowledge and instructor’s concern for learners to predict learner’s perception regarding excellent teaching (Dziuban and Moskal, 2011). Another study gauging students’ satisfaction for online, face-to-face and blended courses was conducted by Dziuban and Moskal (Dziuban et al., 2019). They used image analysis from Guttman’s (Guttman, 1954) and found a single constant across all formats of learning. The study concluded that the students did not differentiate between types of courses from a modalities point of view. Alpha factoring was also used in a study to find out the underlying dimensionality in the level of student satisfaction. This study did so while keeping in view the varying degrees of the ambivalence of the students toward their online courses (Dziuban et al., 2013). The study hypothesized that ambivalent students perhaps were more reflective and thoughtful in evaluating their courses. Keeping in view the above mentioned scientific facts, it is hypothesized that digital readiness has a positive association with e-learning satisfaction.

**H1**: Digital readiness has a positive association with e-learning satisfaction.

### Psychological capital and its role as a mediator between digital readiness and e-learning satisfaction

PsyCap is defined as collective psychological resources that an individual may use to improve their performance to enhance their rate of success (Alessandri et al., 2018). Luthans and his associates define this state of development as having four positive psychological resources. These resources are as follows: self-efficacy, optimism, hope and resilience (Luthans et al., 2004). Research regarding PsyCap has its origin in positive psychology and social learning theory. It takes place mostly in the context of a firm in order to understand the positive forms of motivation and matters of human resource management (Luthans, 2012; Anglin et al., 2018). Psychological capital has been widely and extensively used in research but limited number of studies have been carried out on students and academic settings particularly in the domain of e-learning, online learning and innovative digital learning. Previous research studies which have been carried out on students and PsyCap include Zhao’s study examining PsyCap and the entrepreneurial orientation of students (Zhao et al., 2020). Another research studied the role of PsyCap in shaping the career adaptability of students (Mercan, 2016). Another study attempted to examine how PsyCap played a role in life satisfaction (Kalhori et al., 2018). Few other studies highlighted the relationship between PsyCap and the academic performance of business students (Siu et al., 2014; Margaça et al., 2021). A study found that PsyCap helped keep stress at bay in students (Kaur and Sandhu, 2016). These studies drew a significant picture of the role PsyCap played in shaping academic outcomes. A study concluded that higher self-confidence resulted from higher PsyCap, moreover, PsyCap was found to be enabling individuals to set their goals and improve their professional skills (Hazan Liran and Miller, 2019). Therefore, a study implicitly puts forward that PsyCap can help garner motivation (Simsek and Balaban Sali, 2020). The same is supported by research according to which intrinsic motivation is seen to improve in the wake of higher PsyCap (Liang et al., 2018). Furthermore, training sessions to improve PsyCap also tend to lead to personal and professional development (Liang et al., 2018). Studies have also attempted to examine the relation among sub-factors of both variables. For example, higher self-efficacy can help individuals accumulate a higher level of satisfaction (Jiang, 2021). A significantly positive relation was found between hope and satisfaction among students (Brulde et al., 2018). Most coping strategies depend on hope as one of the main determinants (Liang et al., 2020; Villanueva-Flores et al., 2021). Hope enables students to keep the level of PsyCap high and try to achieve their goals proactively. Resilience and satisfaction have a close link to each other and hope is found to play a mediating role here as well, though, this role is partial. One study holds that readiness has a significant mediating role in the PsyCap of students and their academic engagement (Sava et al., 2020). Readiness plays an important role between PsyCap and academic satisfaction. Therefore, mediation methodology suggests that PsyCap and satisfaction have a deep interconnection (Sánchez-Cardona et al., 2021).

A research study conducted in 2015 has related PsyCap with students’ satisfaction. It has been stated in this study that students can be made to adopt certain positive states related to PsyCap, whereas, the instructor can also use different motivational exercises to help students remain motivated in an online learning environment. Self-efficacy and self-esteem promote the learning satisfaction. This satisfaction enhances the positive psychology of a person and develops psychological capital (Naresh and Reddy, 2015). Having laid out the definitional construct of PsyCap, the study attempts to link it with digital world using literature produced as the result of previous research in this domain (McKenny et al., 2013). New realities of how we learn and how we work are being formed by our digital abilities. There are a number of occupations that are on the verge of being annihilated by the digital revolution (Frey and Osborne, 2017). The voices advocating the importance of digital readiness have increased both in volume and intensity (López Peláez et al., 2020). There have been calls for the development of specific digital literacy learning methods (Campbell and Kapp, 2020; Knight et al., 2020). The importance of digital readiness has increased manifolds (al Seghayer, 2020) as many pieces of research highlighting the fact that digital readiness has a direct association with the trajectory of the academic journey of a student (Bergdahl et al., 2020). There is no denying the importance of new emerging technologies, however, lack of readiness to adopt these innovations remains one of the most difficult challenges (Agyei and Voogt, 2015). This study postulates that positive psychology plays an important role in promoting a digital environment (Sibgatullina et al., 2019). Research studies have shown that positive psychology, self-efficacy and digital literacy enhance the outcomes linked with e-learning (Gaag et al., 2022).

PsyCap has been studied extensively for its role as a mediator between various variables including behaviors, outcomes and satisfaction. Most of the studies have been conducted for workplace and professional organization. A limited number of studies have been conducted on students. Although, digital literacy and e-learning have been extensively studied recently but no study has explored the relationship between digital readiness, e-learning satisfaction and psychological capital. Luthans and coauthors found that PsyCap mediated the relationship between support and efficiency (Luthans, 2012). Murray attempted to examine the mediating role of PsyCap in linking behavior and outcomes (McMurray et al., 2010; Foster, 2020). Leadership behavior and PsyCap were positively associated (McMurray et al., 2010). These particular aspects have been validated by other studies as well (Ravaji, 2016). An individual-level motivation is reckoned important by PsyCap that enhances the learning experience of students. The role of metacognition in online learning can better be understood if PsyCap is recognized as a part of a larger COI framework (Garrison and Akyol, 2015). If the learners are cognitively and motivationally engaged, an individual-level construct can become useful in creating an efficient online learning experience for all (Shea and Bidjerano, 2010). When learners tend to engage in the process of learning, they heavily rely on individual-level motivators. If these motivators are fostered properly chances of having desired outcomes become highly likely. Studies have shown the deep influence of PsyCap on performance at work (Luthans and Stajkovic, 1998; Luthans et al., 2005, 2007b,^2010^; Youssef and Luthans, 2007; Gooty et al., 2009), creativity (Sweetman et al., 2011), personality and well-being of employees. Performance at a workplace or in an academic setting depends on the cognitive abilities of an individual, therefore, it is convenient to suggest that PsyCap can manifest similar effects in an academic environment as it does in a workplace setting. Psychological capital has been studied for its mediating role in an offline learning environment (Chen et al., 2021). Although psychological capital has been studied as a mediator but no study has been done to explore the role of psychological capital as a mediator between digital readiness and e-learning satisfaction, thus it is hypothesized in this study that

**H2**: Digital readiness has a positive association with psychological capital.

**H3**: Psychological capital has a positive association with e-learning satisfaction.

**H4**: Psychological capital mediates the positive association between digital readiness and e-learning satisfaction.

### Mindfulness as a moderator between digital readiness, e-learning satisfaction and psychological capital

According to the American Psychological Association (American Psychological Association, 2012), mindfulness is defined as the ability to have moment-to-moment awareness of one’s experience backed by objectivity (Hafeman et al., 2020). Mindfulness and learning are clubbed together for many proven advantages such as improved performance, enhanced motivation, better physical and mental health, less stress and increased confidence to tackle exam-related pressure (Wang et al., 2016). Mindfulness, in the context of learning, refers to readiness toward changing circumstances, remaining watchful of the new information and implicit awareness of all possible perspectives (Tang et al., 2017). Academic mindfulness can be achieved *via* training the brain by not cutting the clutter and focusing on the most important and pressing scenarios. Practicing mindfulness enhances short as well as long-term memory as suggested by Yeh et al. (2019). They also found a link between mindfulness with creativity too. Enhanced creativity was reported in extended practicing of mindfulness (Yeh et al., 2019). The role of mindfulness has been studied in different fields by researchers such social psychology, health psychology, clinical psychology and health psychology. Mindfulness has been studied and examined quite extensively, however, its impact and role in the context of information-keeping behavior have yet to be studied as vigorously. Studies have found that mindfulness is key to enhancing the psychological strength of a person. It inculcates resilience, satisfaction, bravery and self-esteem (Yeh et al., 2019).

Implied by instructional and motivational theory, when the students are made to actively engage with the learning material, they are more likely to exhibit a deeper understanding of the academic content. Students tend to comprehend and understand the learning material that is presented in an organized manner with a deliberate effort to create a learning environment that encourages engagement. Monotonous and expected methods have been found to be entirely unengaging and have the least impact as far as learning is concerned (Lin, 2020). Multiple research studies have found the unique role of mindfulness in a learning process (Shamas and Maker, 2018; Hensley, 2020). According to studies, it improves cognitive functioning by enhancing cognitive skills (Hensley, 2020). It also improves memory (Niraj et al., 2020), creativity (Hensley, 2020), attention and productivity (Wang and Liu, 2016). In recent studies, moderating role of mindfulness has been studied among the users of various digital platforms (Gu and Hong, 2019). According to studies mindfulness makes sure the harmonious internet use. With the help of mindfulness, the online leaners can seek knowledge consciously and more accurately (Lekkas et al., 2021).

According to social cognitive theory, the self-regulated learning model has three phases of self-reflection, forethought and performance. This model stresses on strategies and motivational factors in online learning. Online learning is a kind of self-regulated learning. It requires students to develop self-directive skills. The nurturing of these skills depends on the level of digital readiness of a student. A student can only mindfully enhance his digital literacy if he or she has a higher level of digital readiness and lower level of resistance to innovation (Carter et al., 2020)PsyCap and mindfulness have been related by various research studies. The studies have found a positive association between these two variables. Mindful practices enhance the psychological capital of individuals (Biswal and Srivastava, 2022). Studies have shown that mindful training increases resilience, and reduce procrastination in the young students (Li and Li, 2020). The moderating role of mindfulness between digital readiness and e-learning satisfaction and psychological capital and e-learning satisfaction has not been studied yet, hence it is hypothesized that

**H5**: Mindfulness moderates the positive association between digital readiness and e-learning satisfaction.

**H6**: Mindfulness moderates the positive association between psychological capital and e-learning satisfaction.

**FIGURE 1:**
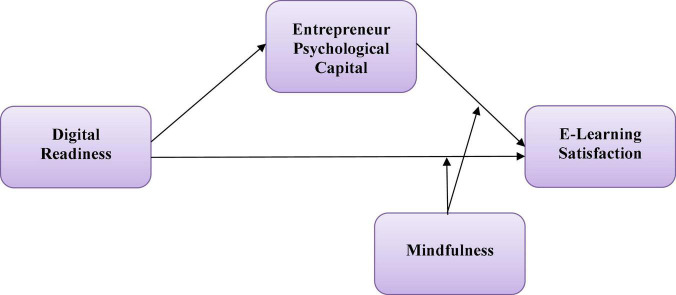
Conceptual framework.

The present study’s conceptual framework is given in Figure 1.

## Research methods

### Study design

For empirical analyses, the present study gathered data from music learning students of entrepreneur training institutes in China. This study considers music learning students for data collection because literature largely ignores them. There is a huge school-based music education system in China and also experienced staff to teach and train the students. Still, there is a need to consider the ways by which the learning satisfaction and performance of students could be increased. Therefore, this study aims to determine how digital readiness affects the E-learning satisfaction of music learning students in China. The author contacted the heads of entrepreneurs training institutes for permission to collect student data. The author got an appointment, visited the entrepreneur training institutes, and had a detailed meeting with them. In the meeting, the author elaborated on the purpose of data collection and satisfied the heads regarding their data confidentiality. The author also explained the present study’s practical importance and ensured that it would be shared with them at their request. Hence, the heads of entrepreneurs’ training institutes showed their consent. This way, the heads allowed the author to data collection. The author developed a cover letter explaining the study data collection objective and the confidentiality of this data for students. The author distributed this cover letter along with the questionnaires. This letter ensured the students that their data would be examined for the present study purpose only, and aggregated outcomes would be publicized as individual-level responses of students would be demolished. The letter also explained the questionnaire answers, such as no answers are measured wrong or right for this study as their actual answers would be useful for the present study results. So, avoid consultation while answering the questionnaires. Following this step, the author ensured to get as natural as possible responses from students. Moreover, this step also proved helpful in boosting the students’ confidence.

For the easiness of the students, the author developed dual-language questionnaires, such as first developed in English and then translated into Chinese. For the translation, the author appointed a language specialist. The author also verified the translated questionnaire from the senior researchers. According to the senior researchers’ suggestion, the author gathered sample-based data to verify the proficiency and easiness of the language. In this way, all the difficulties and errors were revised and finalized the questionnaire for data collection. The senior researchers approved the final questionnaire and allowed to collect data. The author also applied the time lag data approach to collect student data. Following this method, the questionnaires regarding different constructs are distributed at different times. Hence, the present study included a hidden code in the questionnaire to recognize the same respondents. The author distributed 800 questionnaires by following a convenient sampling technique. For the first time, the author distributed questionnaires regarding the independent construct, such as digital readiness, and received 689 questionnaires. By scrutinizing the same respondents and proper filling, the author ensured 633 complete and valid responses in the first wave. After the fifteen-day gap, the author distributed questionnaires regarding the mediator construct, such as entrepreneur psychological capital.

For the second time, the author received 577 complete, valid responses. After another fifteen-day gap, the author gathered data on the dependent variable, e-learning satisfaction. This time author found 403 complete and accurate responses for the next process. The author gathered data on moderate construct mindfulness after the further fifteen-day gap. This time the author collected 376 complete questionnaires by scrutinizing the same respondents and proper filling. Thus, the empirical examination of the present study is based on 376 respondents.

### Measures

This study used five points Likert scale to measure the participants’ responses. This scale consists of five numbers where 1 means “strongly disagree,” 2 means “disagree,” 3 means “neutral,” 4 means “agree,” and 5 means “strongly agree.” This study considered previously validated items to assess the variables. The detail of the items is added in Appendix A.

#### Digital readiness

The construct digital readiness was measured with 10 items scale adopted from Hong and Kim (2018) regarding digital application usage, information-seeking skills, and information-sharing behavior, as these three dimensions were fitted according to our research context. The sample item included, “I can use the fundamental functions of a presentation program (e.g., Microsoft PowerPoint) for class presentations.” The Cronbach’s alpha value is 0.918.

#### Entrepreneur psychological capital

The psychological capital was measured with 24 items scale adapted from Luthans et al. (2007b). The sample item included, “I feel confident analyzing a long-term problem to find a solution.” The Cronbach’s alpha value is 0.954.

#### E-learning satisfaction

The e-learning satisfaction construct was measured with 2 items scale adapted from Szymanski and Richard (2000), Del Barrio-García et al. (2015). The sample item included, “You feel satisfied with your e-learning experience.” The Cronbach’s alpha value is 0.819.

#### Mindfulness

The construct mindfulness was measured with 5 items scale adapted from (Chen and Eyoun, 2021). The sample item included, “It seems I am running on automatic, with much awareness of what I’m doing.” The Cronbach’s alpha value is 0.864.

## Results

### Assessment of measurement and structural model

Structural equation modeling (SEM) is considered one of the most appropriate statistical models for data analyses. Covariance-based (CB-SEM) and variance-based partial least squares structural equation modeling (PLS-SEM) are two different types of SEM (Hair et al., 2019a). The main difference in both methods is that CB-SEM is considered for theory acceptance and rejection, while PLS-SEM is considered for advancing and developing the theories (Hair et al., 2016; Bashir et al., 2021). The present study applied the PLS-SEM technique for data analysis. The key rationale behind this selection is the usefulness of PLS-SEM for both confirmatory and exploratory studies (Hair et al., 2011). PLS-SEMi s a useful approach for complex and multi-orders-based models and needs no specific data normality conditions. PLS-SEM is also appropriate for evaluating small data sets (Hair et al., 2016). Therefore, this study considers the PLS-SEM method for empirical data analyses using Smart PLS 3.3.3 software. The outcomes of PLS-SEM-based analysis are evaluated in two stages, including model measurement and structural model evaluation. The measurement model stage assesses the reliability and validity of the constructs, whereas the structural model investigates the relationship between the proposed hypotheses. The acceptance or rejection of a hypothesis is evaluated through the “t” statistic and “p” values.

The model measurement outcomes are comprised of two parts: model reliability and validity. The present study considered the values of “Cronbach’s alpha, roh-A, composite reliability, and average variance extract (AVE)” to authenticate the model’s reliability (Hair et al., 2016), and all values are shown in Table 1. The values of Cronbach’s alpha are accepted if they are larger than 0.7 (Hair et al., 2017). Similarly, the value of composite reliability should also be greater than 0.7. The Cronbach’s alpha values of models’ constructs (digital readiness, e-learning satisfaction, mindfulness, and psychological capital) are 0.918, 0.819, 0.864, 0.954, and the composite reliability values of models’ constructs are 0.930, 0.917, 0.901, and 0.958, respectively. All Cronbach’s alpha and composite reliability values are according to acceptable criteria, validating the model’s reliability. The values of roh-reliability 0.929, 0.819, 0.879, and 0.955 are also according to the acceptable criteria (Hair et al., 2016). The average variance extracts (AVE) values greater than 0.5 are considered ideal for the convergent validity of the model. The Table 1 shows that the AVE values of all constructs (0.572, 0.847, 0.645, and 0.521) are according to acceptable criteria.

**TABLE 1:** Outer loadings reliability and convergent validity of the study constructs.

Construct	Item	Outer loadings	VIF	Alpha	roh-A	Composite Reliability	AVE
DR	DR1	0.768	2.185	0.918	0.929	0.930	0.572
	DR2	0.719	1.887				
	DR3	0.773	2.354				
	DR4	0.827	3.046				
	DR5	0.805	2.628				
	DR6	0.780	2.263				
	DR7	0.709	2.239				
	DR8	0.701	2.414				
	DR9	0.715	2.119				
	DR10	0.756	2.263				
ELS	ELS1	0.921	1.925	0.819	0.819	0.917	0.847
	ELS2	0.919	19.25				
MF	MF1	0.810	2.533	0.864	0.879	0.901	0.645
	MF2	0.825	3.302				
	MF3	0.823	3.411				
	MF4	0.765	2.139				
	MF5	0.792	2.197				
PC	PC1	0.764	2.524	0.954	0.955	0.958	0.521
	PC3	0.686	2.469				
	PC4	0.763	3.203				
	PC5	0.709	2.613				
	PC6	0.745	2.604				
	PC7	0.713	2.368				
	PC8	0.744	2.601				
	PC9	0.729	2.742				
	PC10	0.752	2.875				
	PC11	0.704	2.690				
	PC12	0.742	3.264				
	PC13	0.750	3.693				
	PC14	0.752	3.083				
	PC15	0.739	3.213				
	PC16	0.709	2.769				
	PC17	0.658	2.403				
	PC18	0.714	2.568				
	PC19	0.730	2.639				
	PC22	0.699	2.790				
	PC23	0.691	2.852				
	PC24	0.643	2.570				

DR, digital readiness; ELS, e-learning satisfaction; MF, mindfulness; PC, psychological capital.

**FIGURE 2:**
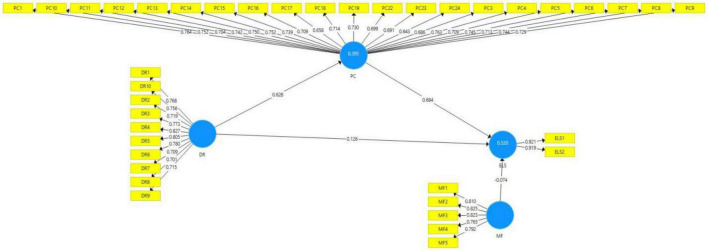
Path estimates and outer loadings.

Table 1 explains that the present study’s framework is based on 38 items of the four variables. All items’ outer loading values of models’ constructs are shown in Table 1. The outer loading values of items are considered reliable if they are greater than 0.7 (Hair et al., 2017). Figure 2 depicts that the outer loading values of all items are according to the required criteria, except for items PC2, PC20, and PC1. Hence, these items were deleted for better reliability outcomes. The items (PC3, PC17, PC22, PC23, and PC24) below 0.7 are retained because these items did not affect the AVE value. The VIF values are assessed to validate the collinearity issues in the model. The model is considered free from collinearity issues if the VIF values are below 0.5 (Hair et al., 2019a). According to the results in Table 1, all VIF values are less than 0.5, such as the variable “psychological capital” item PC-13 has the highest VIF value (3.693). Hence, it is verified that the model of the present study is free from collinearity issues.

The R^2^ values are evaluated to define the model’s strength, such as the values of latent variables greater than or near 0.5 specifying moderate strength of the model, and the values near 0.25 showing weak model strength (Hair et al., 2014). The R^2^ values of the present study’s model’s endogenous variables (psychological capital and e-learning satisfaction) are 0.395 and 0.520, respectively, which shows moderate model strength (Hair et al., 2017). The model’s cross-validated redundancy (Q2) values are considered significant if they are greater than zero (Hair et al., 2014). The Q^2^ values of all latent variables of the current study are greater than zero, which is another positive indication of model significance.

Fornell–Larcker criterion and heterotrait–monotrait (HTMT) ratios are well-known approaches that measure the discriminant validity of the model constructs (Hair et al., 2017). The present study used these two approaches for assessing constructs’ validity. The Fornell-Larcker criterion is measured by taking the square roots of AVE values of model constructs (Hair et al., 2014). The Fornell–Larcker criterion values of constructs are presented in Table 2. The values under the Fornell–Larcker criterion are accepted if each column’s upper side first value is higher than the below values. Table 2 shows that all values of the Fornell–Larcker criterion are as per the accepted criteria. Thus, this study model confirms that discriminant validity established on the Fornell-Larcker criterion has been achieved. In addition, according to the specified criteria, the HTMT values of all variables should be less than 0.85; however, values greater than 0.90 are also acceptable (Hair et al., 2019b). According to the outcomes (Table 3), the HTMT values of constructs are less than 0.85, which confirms that discriminant validity in the current study’s model had been achieved.

**TABLE 2:** Discriminant validity (Fornell-Larker-1981 Criteria).

Construct	DR	ELS	MF	PC
DR	***0.757***			
ELS	0.524	***0.920***		
MF	0.432	0.445	***0.803***	
PC	0.628	0.713	0.680	***0.722***

DR, digital readiness; ELS, e-learning satisfaction; MF, mindfulness; PC, psychological capital. The bold values indicates the results for corresponding statistics.

**TABLE 3:** Discriminant validity (HTMT).

Construct	DR	ELS	MF	PC
DR	–	–	–	–
ELS	0.565	–	–	–
MF	0.469	0.503	–	–
PC	0.653	0.796	0.725	–

DR, digital readiness; ELS, e-learning satisfaction; MF, mindfulness; PC, psychological capital.

### Hypotheses testing

The present study’s empirical investigation is conducted using 5,000 samples of the bootstrapping method (Hair et al., 2014, 2016). The outcomes of the direct, indirect, and total paths are depicted in Table 4. The present study considered the “t” values and “p” values of statistics for the acceptance and rejection of hypotheses. Table 5 shows the results of the hypotheses proposed by the present study. The outcomes of hypothesis 1 (*t* = 2.972, *p* = 0.003) confirmed that digital readiness positively correlates with e-learning satisfaction. Additionally, the beta value of hypothesis 1 confirmed that one unit change in the independent variable (digital readiness) would result in 0.157 changes in the dependent variable (e-learning satisfaction). Hence hypothesis 1 of the present study is accepted. The findings of the second hypothesis (*t* = 10.443, *p* = 0.000) confirmed that digital readiness has a positive association with psychological capital. Additionally, the beta value of H2 depicted that one unit change in the independent variable (digital readiness) would result in 0.628 changes in the dependent variable (psychological capital). Hence the second hypothesis of the present study is also accepted. The findings of the third hypothesis (*t* = 9.228, *p* = 0.000) confirmed that psychological capital has a positive association with e-learning satisfaction. Moreover, the beta value indicated that one unit change in the independent variable (psychological capital) would result in 0.720 changes in the dependent variable (e-learning satisfaction). Hence the H3 of the present study is also accepted.

**TABLE 4:** Direct, indirect, and total path estimates.

Direct path	Beta	SD	t	p
DR - > ELS	0.157	0.053	2.972	0.003
DR - > PC	0.628	0.060	10.443	0.000
MF - > ELS	−0.038	0.055	0.690	0.490
MF*DR - > ELS	−0.112	0.058	1.928	0.054
MF*PC - > ELS	0.124	0.054	2.302	0.021
PC - > ELS	0.720	0.078	9.228	0.000

**Indirect Path**	**Beta**	**SD**	**t**	**p**

DR - > PC - > ELS	0.452	0.079	5.075	0.000

**Total Path**	**Beta**	**SD**	**t**	**p**

DR - > ELS	0.609	0.082	7.408	0.000
DR - > PC	0.628	0.060	10.443	0.000
MF - > ELS	−0.038	0.055	0.690	0.490
MF*DR - > ELS	−0.112	0.058	1.928	0.054
MF*PC - > ELS	0.124	0.054	2.302	0.021
PC - > ELS	0.720	0.078	9.228	0.000

DR, digital readiness; ELS, e-learning satisfaction; MF, mindfulness; PC, psychological capital.

**TABLE 5:** Hypotheses testing.

Hypotheses		Coefficient (Beta)	S.D	*t*	*p*	Status
H1	DR - > ELS	0.157	0.053	2.972	0.003	Supported
H2	DR - > PC	0.628	0.060	10.443	0.000	Supported
H3	PC - > ELS	0.720	0.078	9.228	0.000	Supported

	**Mediation Hypotheses**	**Coefficient (Beta)**	**S.D**	***t***	***p***	**Status**

H4	DR - > PC - > ELS	0.452	0.079	5.705	0.000	Supported

	**Moderation Hypotheses**	**Coefficient (Beta)**	**S.D**	***t***	***p***	**Status**

H5	MF*DR - > ELS	−0.112	0.058	1.928	0.054	Not Supported
H6	MF*PC - > ELS	0.124	0.054	2.302	0.021	Supported

DR, digital readiness; ELS, e-learning satisfaction; MF, mindfulness; PC, psychological capital.

The present study also assumes the mediating role of psychological capital in the relationship between digital readiness and e-learning satisfaction. For the empirical investigation of mediating role, the present study assumes H4. According to findings (*t* = 5.705, *p* = 0.000), psychological capital positively mediates the association between digital readiness and e-learning satisfaction, and the path value of H4 is (0.452). Hence, it is confirmed that the fourth hypothesis of the present study is accepted.

The present study also evaluated the moderating role of mindfulness in the relationship between digital readiness and e-learning satisfaction and the relationship between psychological capital and e-learning satisfaction, respectively. For empirical investigation present study assumes H5 and H6. The results of H5 (*t* = 1.928, *p* = 0.054) revealed that mindfulness does not moderate the relationship between digital readiness and e-learning satisfaction. Therefore, the fifth hypothesis of the present study is rejected. The outcomes of H6 (*t* = 2.302, *p* = 0.021) confirmed that mindfulness positively moderates the relationship between psychological capital and e-learning satisfaction. Hence, the sixth hypothesis of the present study is accepted.

## Discussion

In this digital era, innovative technologies are a valuable source of increased work efficiencies and productivity (Ergun and Adibatmaz, 2020). Organizations are seeking ways to enhance their work productivity by adopting innovative technologies. The education institutions are also consistently concerned with boosting students’ academic efficacy through innovative technologies. The students are considered digital natives and are expected to have higher e-learning competencies to improve their academic effectiveness (Fernandez et al., 2022). However, digital readiness is an important factor that can play a valuable role in boosting students’ e-learning abilities and satisfaction (Kim et al., 2019). The previous studies of students’ e-learning abilities revealed the lack of students’ digital readiness for academic achievements. Therefore, the present study aims to examine the role of digital readiness in the e-learning satisfaction of students. For empirical investigation, this study proposes six hypotheses. First, the current study hypothesized that digital readiness positively correlates with e-learning satisfaction. According to the second hypothesis, digital readiness positively impacts one-learning satisfaction. Third, the current study assumes that psychological capital positively correlates with e-learning satisfaction. For determining the mediating role, this hypothesized that psychological capital mediates the association between digital readiness and e-learning satisfaction. This study also attempts to check the moderating role of mindfulness in the relationship between digital readiness and e-learning satisfaction and the relationship between psychological capital and e-learning satisfaction.

The findings of the present study revealed that digital readiness has a positive association with e-learning satisfaction, which means the first hypothesis of this study is accepted. These findings are consistent with prior studies (Ciucan-Rusu et al., 2020; Händel et al., 2020; Osman et al., 2021). According to these studies, digital readiness is an important aspect of increasing the e-learning competencies of students. Moreover, digital readiness is a positive signal and is a motivator for increasing the work productivity of students. According to Kim et al. (2019), digital readiness enhances students’ technology-related knowledge, skills, and competencies, which can play a constructive role in meeting their academic expectations. The findings of this study further acknowledged that digital readiness has a positive association with psychological capital. These findings are consistent with prior studies (Harris et al., 2020; Luo et al., 2022), as these studies also give arguments about the positive role of readiness in enhancing students’ psychological capital. The findings also confirmed that psychological capital positively correlates with e-learning satisfaction. These outcomes are consistent with previous studies’ findings (Harwood and Hassiotis, 2014; Ahmed et al., 2021). According to these studies, students’ online psychological capital can develop their online behavioral engagement and competencies, which could positively increase their e-learning satisfaction.

The present study also assessed the mediating role of psychological capital in the relationship between digital readiness and e-learning satisfaction. The results confirmed that the fourth hypothesis is accepted, which means that psychological capital positively mediates the association between digital readiness and e-learning satisfaction. The present study also assumes the moderating role of mindfulness in the relationship between digital readiness and e-learning satisfaction and the relationship between psychological capital and e-learning satisfaction. The findings revealed that the fifth hypothesis is rejected, meaning mindfulness does not moderate the relationship between digital readiness and e-learning satisfaction. However, the findings further acknowledged that mindfulness moderates the association between psychological capital and e-learning satisfaction.

## Theoretical and practical implications

The study suggests that an integrated approach is required to be implemented at universities for both online and offline learning settings. Furthermore, the study suggests having more opportunities be provided to the students to get accustomed to the e-learning tools and environments so that they can fully benefit from its utility. The academic institution needs to develop comprehensive training programs to teach the correct and effective use of e-learning platforms (Luthans and Stajkovic, 1998). Furthermore, a follow-up program should aim at learning through the individual feedback of the students so that the training modules can be altered and bettered according to individuals’ needs. Extra-curricular engagements are suggested to be used as a medium to normalize and advance the cause of e-learning. General education on the integration of technology with academic routines can be imparted through regular workshops and relevant courses.

There is also a need for the faculty to recognize that technology integration in the courses is paramount and there has to be an effort to make this integration possible in an effective way. The e-learning environment at the campus must be designed in a way to helps learners achieve better academic outcomes. The design of the e-learning system should be user-friendly and should only facilitate learning and not make it cumbersome. Despite the fact the younger generation is generally considered digital natives, however, there still remains that need to have a technology-driven e-learning setup with the potential to enhance the learning ability of these digital native students. The satisfaction of the learners vis-à-vis e-learning modalities is of paramount importance. Students’ feedback is essential in ensuring that the integration of technology and academic endeavors are garnering desired outcomes and that students are satisfied with this integration. Blended learning modules that involve teacher-led instructions and technology-enabled pedagogical plans and content have proven to be highly effective in enhancing the learning experience of the students. The utilization of mindfulness and PsyCap in the classrooms can significantly improve the learning experience of the students, furthermore, their satisfaction with e-learning will also enhance.

## Limitations

The present study serves the literature in multiple ways, but still, there are some limitations. These gaps may become opportunities for scholars to conduct their research in the future. First, this study is conducted using a small sample size; in the future, researchers may broaden the sample size to authenticate the present study’s model. Second, this study is conducted in China, and the results may not be generalizable to other contexts. Scholars in the future may conduct the same study in other developing or developed countries for a better understanding of the study model. Third, this study collected the data using a structured questionnaire method; in the future, scholars may consider other data collection methods such as semi-structured questionnaires, interview methods, etc. Fourth, the present study assumes digital readiness as an antecedent of e-learning satisfaction; future studies may consider other possible antecedents like digital innovation and e-learning attitude. Fifth, this study examined the mediating role of psychological capital in the relationship between digital readiness and e-learning satisfaction; future studies may consider other possible mediating variables like e-learning adoption, etc. Finally, this study assumes moderating role of mindfulness in the relationship between digital readiness and e-learning satisfaction and the relationship between psychological capital and e-learning satisfaction. Future researchers may consider other moderators for this study, like emotional intelligence and psychological engagement.

## Conclusion

The e-learning abilities of students are an important source of enhancing their academic effectiveness. Digital readiness is an important factor that can play a valuable role in boosting students’ e-learning abilities and satisfaction. Therefore, the present study aims to examine the association of digital readiness with the e-learning satisfaction of students. Based on the theory of motivation, the present study hypothesized that digital readiness positively correlates with e-learning satisfaction and psychological capital. This study also aims to examine the relationship of psychological capital with e-learning satisfaction. The present study assesses the mediating role of psychological capital in the relationship between digital readiness and e-learning satisfaction. This study also proposes the moderating role of mindfulness in the relationship between digital readiness and e-learning satisfaction and the relationship between psychological capital and e-learning satisfaction, respectively. The present study confirmed that digital readiness positively correlates with e-learning satisfaction and psychological capital. The findings further acknowledged that psychological capital positively enhances e-learning satisfaction. The results also confirmed that psychological capital mediates the association between digital readiness and e-learning satisfaction. However, the results revealed that mindfulness does not moderate the association between digital readiness and e-learning satisfaction. Additionally, the findings acknowledged that mindfulness moderates the relationship between psychological capital and e-learning satisfaction.

## Data availability statement

The original contributions presented in the study are included in the article/supplementary material, further inquiries can be directed to the corresponding author/s.

## Author contributions

YH conceived the idea, designed, and wrote the manuscript.

## Appendix A

### Digital readiness

#### Digital application Usage

1. I can use the fundamental functions of a presentation program (e.g., Microsoft PowerPoint) for class presentations.
2. I can use the fundamental functions of word-processing programs to create and edit documents for class assignments.
3. I can use spreadsheet programs (e.g., Microsoft Excel) to handle data and analyze it for class assignments.

#### Information-seeking skills

4. I can use a variety of available options to search for information that my colleagues are not aware of.
5. I can inform my classmates of different ways to effectively search for information.
6. I can generate keywords to search for information for academic work.

#### Information-sharing behavior

7. I can interact with classmates using real-time communication tools, for example, video conferencing tools or messengers.
8. I can share my opinions online, for example, with blogs, social networking services, or web pages.
9. I can share my files with classmates using online software.
10. I can collaborate with classmates using online software.

### Entrepreneur psychological capital

1. I feel confident analyzing a long-term problem to find a solution.
2. I feel confident in representing my work area in meetings with management.
3. I feel confident contributing to discussions about the company’s strategy.
4. I feel confident helping to set targets/goals in my work area.
5. I feel confident contacting people outside the company (e.g., suppliers, customers) to discuss problems.
6. I feel confident presenting information to a group of colleagues.
7. If I should find myself in a jam at work, I could think of many ways to get out of it.
8. At the present time, I am energetically pursuing my work goals.
9. There are lots of ways around any problem.
10. Right now I see myself as being pretty successful at work.
11. I can think of many ways to reach my current work goals.
12. At this time, I am meeting the work goals that I have set for myself.
13. When I have a setback at work, I have trouble recovering from it, moving on (R).
14. I usually manage difficulties one way or another at work.
15. I can be “on my own,” so to speak, at work if I have to.
16. I usually take stressful things at work in stride.
17. I can get through difficult times at work because I’ve experienced difficulty before.
18. I feel I can handle many things at a time at this job.
19. When things are uncertain for me at work, I usually expect the best.
20. If something can go wrong for me work-wise, it will (R).
21. I always look on the bright side of things regarding my job.
22. I’m optimistic about what will happen to me in the future as it pertains to work.
23. In this job, things never work out the way I want them to (R).
24. I approach this job as if “every cloud has a silver lining.”

### E-learning satisfaction

1. You feel satisfied with your e-learning experience.
2. You feel pleased during your e-learning experience.

### Mindfulness

1. It seems I am running on automatic, without much awareness of what I’m doing.
2. I get so focused on the goal I want to achieve that I lose touch with what I’m doing right now to get there.
3. I rush through activities without being really attentive to them.
4. I do jobs or tasks automatically, without being aware of what I’m doing.
5. I find myself doing things without paying.
